# The tyrosine-kinase inhibitor Nintedanib ameliorates autosomal-dominant polycystic kidney disease

**DOI:** 10.1038/s41419-021-04248-9

**Published:** 2021-10-14

**Authors:** Abeda Jamadar, Sreenath M. Suma, Sijo Mathew, Timothy A. Fields, Darren P. Wallace, James P. Calvet, Reena Rao

**Affiliations:** 1 The Jared Grantham Kidney Institute, University of Kansas Medical Center, Kansas City, KS USA; 2 Department of Medicine, University of Kansas Medical Center, Kansas City, KS USA; 3 Department of Pharmaceutical Sciences, School of Pharmacy, North Dakota State University, Fargo, ND USA; 4 Department of Pathology and Laboratory Medicine, University of Kansas Medical Center, Kansas City, KS USA; 5 Department of Biochemistry and Molecular Biology, University of Kansas Medical Center, Kansas City, KS USA

**Keywords:** Growth factor signalling, Polycystic kidney disease

## Abstract

Autosomal-dominant polycystic kidney disease (ADPKD) is the most common inherited kidney disease and is characterized by progressive growth of fluid-filled cysts. Growth factors binding to receptor tyrosine kinases (RTKs) stimulate cell proliferation and cyst growth in PKD. Nintedanib, a triple RTK inhibitor, targets the vascular endothelial growth-factor receptor (VEGFR), platelet-derived growth-factor receptor (PDGFR), and fibroblast growth-factor receptor (FGFR), and is an approved drug for the treatment of non-small-cell lung carcinoma and idiopathic lung fibrosis. To determine if RTK inhibition using nintedanib can slow ADPKD progression, we tested its effect on human ADPKD renal cyst epithelial cells and myofibroblasts in vitro, and on *Pkd1*^*f/f*^*Pkhd1*^*Cre*^ and *Pkd1*^*RC/RC*^, orthologous mouse models of ADPKD. Nintedanib significantly inhibited cell proliferation and in vitro cyst growth of human ADPKD renal cyst epithelial cells, and cell viability and migration of human ADPKD renal myofibroblasts. Consistently, nintedanib treatment significantly reduced kidney-to-body-weight ratio, renal cystic index, cystic epithelial cell proliferation, and blood-urea nitrogen levels in both the *Pkd1*^*f/f*^*Pkhd1*^*Cre*^ and *Pkd1*^*RC/RC*^ mice. There was a corresponding reduction in ERK, AKT, STAT3, and mTOR activity and expression of proproliferative factors, including Yes-associated protein (YAP), c-Myc, and Cyclin D1. Nintedanib treatment significantly reduced fibrosis in *Pkd1*^*RC/RC*^ mice, but did not affect renal fibrosis in *Pkd1*^*f/f*^*Pkhd1*^*Cre*^ mice. Overall, these results suggest that nintedanib may be repurposed to effectively slow cyst growth in ADPKD.

## Introduction

In PKD kidneys, cysts develop from the renal tubules and progressively enlarge due to cell proliferation and fluid secretion by tubular epithelial cells [1]. Progressive renal fibrosis accompanies cyst expansion in all forms of PKD, irrespective of the underlying gene mutation, and along with inflammation, could be a final common pathway to end-stage renal disease (ESRD) [2, 3].

The RTK family consists of 58 known transmembrane receptors that regulate cell proliferation, differentiation, migration, metabolism, fibrosis, and angiogenesis. High-affinity binding to ligands such as growth factors, cytokines, or hormones leads to activation and consequent autophosphorylation of RTKs. This, in turn, leads to the recruitment of a wide range of proteins that propagate cell-signaling pathways, including JAK2/STAT, RAS/RAF/MEK/ERK (MAPK), PI3 kinase, AKT, or mTOR signaling.

Under normal physiologic conditions, the activity of RTKs is tightly regulated. However, in various human diseases, aberrant RTK activity occurs due to gain-of-function mutations in their ligand-binding or kinase domains, overexpression of RTKs by genomic amplification, overproduction of ligands, or by autocrine activation of RTKs [4]. Multiple small-molecule inhibitors of RTKs have been developed to specifically target the ATP-binding site of the intracellular tyrosine kinase domain. Moreover, FDA-approved small-molecule inhibitors and monoclonal antibodies that interfere with RTK activation are currently used for the treatment of cancer and lung disease [5, 6].

In PKD, two decades of research have shown that RTKs play an important role in disease progression. The main RTKs that were studied in PKD are EGFR, HER2, and VEGFR. Inhibition of EGFR and VEGFR was shown to slow cyst growth in various PKD rodent models [7–13]. Moreover, tesevatinib, an inhibitor of EGFR, HER2, c-SRC, and VEGFR, which reduced cyst growth in autosomal-recessive PKD (ARPKD) mice [10], was recently tested for efficacy and safety in a clinical phase-2 study in ADPKD patients (ClinicalTrials.gov Identifier: NCT03203642).

Nintedanib (BIBF 1120, Vargatef^®^, Ofev^®^), is a potent small-molecule RTK inhibitor that targets PDGFRα and β, FGFR1,2 and 3, and VEGFR1,2 and 3 [14]. Competitive binding of nintedanib to the ATP-binding pocket of RTKs interferes with cross-autophosphorylation of the receptor homodimers, thereby blocking the signaling cascade. Importantly, nintedanib has undergone extensive human trials [5, 15, 16] and is currently approved by the US FDA for therapy for idiopathic pulmonary fibrosis, interstitial lung disease associated with systemic sclerosis, and chronic fibrosing interstitial lung diseases, and by the European Medicines Agency for non-small-cell lung carcinoma. Preclinical studies have demonstrated nintedanib’s antiproliferative and antitumor effects in various cancers, including kidney, ovarian, pancreatic, colorectal, prostate, and non-small-cell lung cancers [14, 17]. In addition, Nintedanib has been shown to be a potent antifibrotic agent that stimulates promatrix metalloproteinases-2 expression and inhibits tissue inhibitor of metalloproteinases-2 (TIMP2) expression, a combination that promotes extracellular matrix (ECM) degradation in the lungs [18]. Nintedanib treatment also inhibited fibrosis in various cancers and in chronic disease of the lungs and liver [14, 19–21]. Importantly, nintedanib treatment significantly reduced renal fibrosis in unilateral ureteric obstruction and folic acid induced chronic kidney disease in mice [22]. However, the effect of nintedanib has not been tested in PKD. Here we examined the effects of nintedanib on cyst growth and fibrosis in two rapid and slow progressive ADPKD mouse models. The results of these studies are presented.

## Results

### Nintedanib suppressed ADPKD cyst epithelial cell proliferation and cyst growth in vitro

**Fig. 1:**
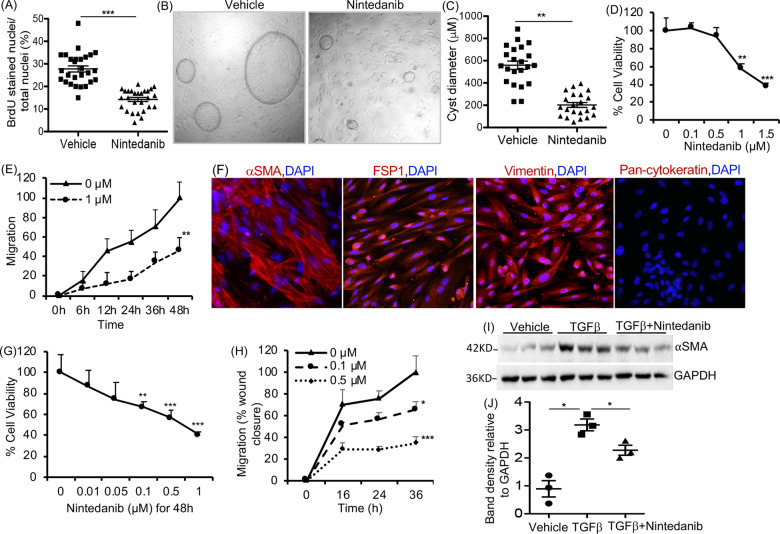
Effect of Nintedanib treatment on ADPKD renal cystic epithelial cells and myofibroblasts. **A** BrdU-incorporation assay in human ADPKD renal cystic epithelial cells treated with nintedanib (1.5 µM for 24 h). *n* = 4 biological replicates (patient samples) and seven technical replicates each. **B** Images of microcyst growth in 3D collagen matrix by human ADPKD renal cystic epithelial cells treated with vehicle or nintedanib (1.5 µM) for 12 days. 1X magnification, and (**C**) quantitation of cyst diameter. *n* = 4 biological replicates and 5 or 6 technical replicates each. **D** Human ADPKD renal myofibroblasts treated with nintedanib for 48 h. MTT assay and **E** migration (% wound closure in a scratch assay) in the presence of 0 or 1 µM nintedanib. *n* = 4 biological replicates and three technical replicates each for **D** and **E**. **F** Human renal ADPDK myofibroblasts immunostained for αSMA, FSP1, vimentin, or pan-cytokeratin. **G** NRK-49F rat renal fibroblasts treated with nintedanib for 48 h. MTT assay. *n* = 7. **H** Migration of NRK-49F cells treated with nintedanib *n* = 4. **I** Immunoblot of NRK-49F cells incubated with TGFβ (2 ng/ml) and nintedanib (1.5 μM) for 48 h and (**J**) quantitation of band density. *P < 0.05 **P < 0.01, ***P < 0.001, vs vehicle or 0 µM nintedanib.

To test the effect of nintedanib on epithelial cell proliferation, primary culture human ADPKD renal cyst epithelial cells were treated with nintedanib for 24 h and cell proliferation was determined by BrdU incorporation. Nintedanib treatment at 1.5 µM dose reduced cell proliferation by over 40% compared with vehicle treatment (Fig. 1A). However in primary culture normal human kidney (NHK) epithelial cells or M1 mouse-collecting duct cells, at 1.5 µM dose, nintedanib did not significantly affect cell proliferation and even at 3 µM, nintedanib reduced proliferation by only 22 and 15%, respectively (Supplemental 1A, B). Nintedanib also reduced cyst growth by human ADPKD renal epithelial cells seeded within a 3D collagen matrix (Fig. 1B) by 5-fold when compared with vehicle-treatment group (Fig. 1C).

### Nintedanib reduced myofibroblast-cell viability and migration in vitro

Nintedanib significantly reduced cell viability (Fig. 1D) and migration (Fig. 1E) of primary-culture myofibroblasts isolated and cultured from human ADPKD kidneys, compared with vehicle. These cells expressed αSMA, vimentin, and fibroblast-specific protein-1 (FSP-1), markers for differentiated myofibroblasts, mesangial cell, and fibroblast, respectively, but not pan-cytokeratin, an epithelial cell marker (Fig. 1F). The αSMA expression in human ADPKD myofibroblasts was not affected by nintedanib at doses that reduced their migration and cell proliferation (Supplemental 1C, D). However, in NRK-49F rat renal fibroblasts, nintedanib treatment not only dose-dependently reduced cell viability (Fig. 1G) and migration (Fig. 1H), but also reduced TGFβ-induced fibroblast-to-myofibroblast differentiation indicated by reduced αSMA levels (Fig. 1I, J).

### Nintedanib treatment reduced renal cystic epithelial cell proliferation and cyst growth in *Pkd1*^RC/RC^ mice

**Fig. 2:**
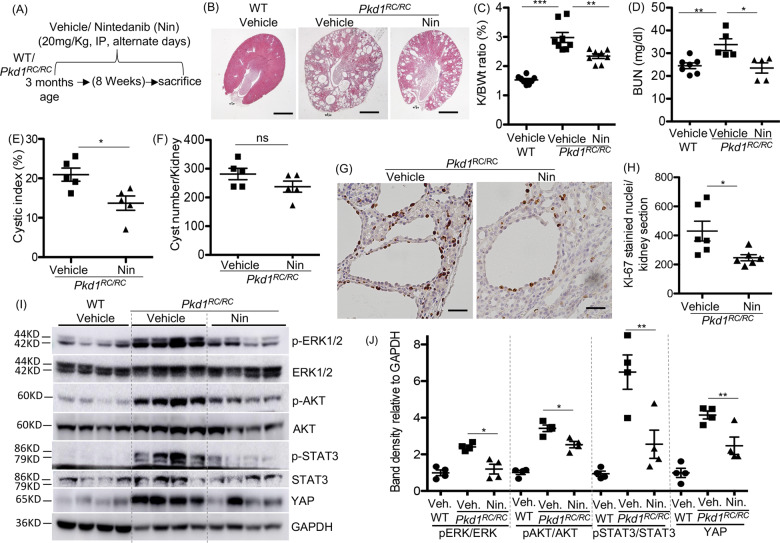
Nintedanib (Nin) treatment reduced renal cyst growth and cell proliferation in *Pkd1*^*RC/RC*^ mice. **A** Scheme of treatment. WT and *Pkd1*^RC/RC^ mice were treated with vehicle or Nintedanib (20 mg/Kg BWt) by IP injection on alternate days from 3 to 5 months of age. **B** H&E staining of kidney sections. Scale bar = 1 mm. **C** Two kidney to body-weight ratios (%). **D** Blood-urea nitrogen (BUN) levels. **E** Cystic index (%), (**F**) Cyst number. **G** Ki-67 immunostaining (scale bar = 50 µM), and (**H**) quantitation of Ki-67-stained nuclei in kidney sections, *n* = 5. **I** Immunoblot of kidney tissue and (**J**) quantitation of band density. *P < 0.05, **P < 0.01, ***P < 0.001 by *T*-test.

In five-month old *Pkd1*^RC/RC^ mouse kidneys, significant increase in protein levels of PDGFRβ and FGFR1, but not VEGFR2, was observed when compared with WT kidneys (Supplemental 2A, B). Both PDGFRα and PDGFRβ mRNA levels were increased in *Pkd1*^RC/RC^ kidneys, when compared with WT kidneys (Supplemental 2C). In *Pkd1*^RC/RC^ mice, nintedanib treatment (20 mg/kg body weight) on alternate days from three months to five months of age (Fig. 2A) significantly reduced kidney size (Fig. 2B), kidney to body-weight ratio (Fig. 2C), blood urea nitrogen (BUN) levels (Fig. 2D), and cystic index (percent cystic area) (Fig. 2E), but not cyst number when compared with vehicle treatment (Fig. 2F). Cystic epithelial cell division indicated by Ki-67 staining was significantly reduced by 42% in nintedanib-treated *Pkd1*^RC/RC^ kidneys, when compared with vehicle treatment (Fig. 2G, H). In WT mice, nintedanib treatment did not significantly affect renal morphology, kidney to body-weight ratio, or cell proliferation when compared with vehicle treatment (Supplemental 3A–D).

### Nintedanib reduced RTK cell signaling in *Pkd1*^RC/RC^ mouse kidneys

We examined the effect of nintedanib on intracellular RTK cell-signaling components, including ERK, AKT, and STAT3 activity, which also regulate cell proliferation, inflammation, and fibrosis in ADPKD kidneys [9, 11, 23]. When compared with WT kidneys, *Pkd1*^RC/RC^ kidneys showed significant increases in pERK1/2 /ERK1/2, pAKT/AKT, and pSTAT3/STAT3 ratios (Fig. 2I, J), suggesting increased activity, which was significantly reduced by nintedanib. YAP, an important Hippo signaling component and mediator of RTK signaling in cancer cells [24], promotes cyst growth [25, 26] and fibrosis [26] in ADPKD. In *Pkd1*^RC/RC^ kidneys, we found significantly increased YAP levels when compared with WT kidneys, and nintedanib treatment significantly reduced YAP levels in *Pkd1*^RC/RC^ kidneys (Fig. 2I, J). However, similarly increased cyclin D1 and c-myc expression in *Pkd1*^RC/RC^ kidneys were not significantly altered by nintedanib treatment (Supplemental 4A, B).

### Nintedanib treatment reduced fibrosis and myofibroblast population in *Pkd1*^RC/RC^ mice

**Fig. 3:**
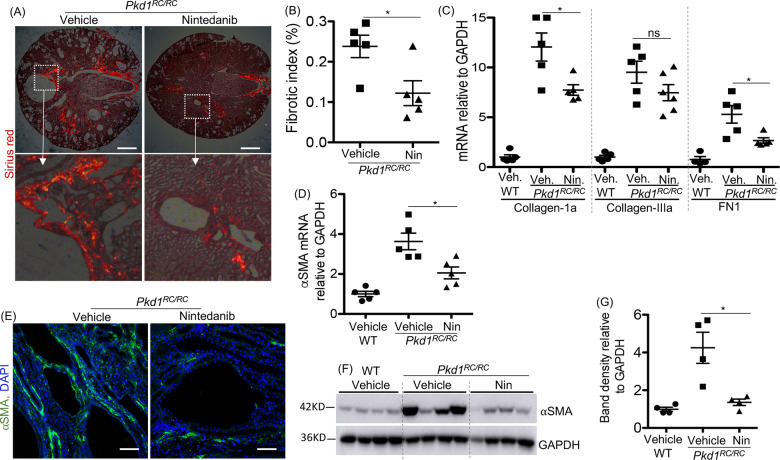
Nintedanib (Nin) treatment reduced fibrosis in *Pkd1*^*RC/RC*^ mice. **A** Sirius red-stained tissue images under polarized light (scale bar = 1 mm) and (**B**) quantitation of fibrosis based on Sirius red staining. **C** Renal mRNA levels for ECM proteins. **D** Renal αSMA mRNA levels. **E** Immunostaining for αSMA (green) in mouse kidney tissue (scale bar = 50 µM). **F** Immunoblot for αSMA in kidney tissue and (**G**) quantitation of band density. *P < 0.05, **P < 0. 01, ***P < 0.001 by *T*-test.

Renal fibrosis in *Pkd1*^RC/RC^ kidneys indicated by Sirius Red staining and quantitation was significantly reduced in the nintedanib-treated *Pkd1*^RC/RC^ kidneys (Fig. 3A, B). Nintedanib also reduced mRNA levels of ECM proteins such as collagen-1a and fibronectin (FN1), but not collagen-IIIa (Fig. 3C). High αSMA mRNA and protein levels (Fig. 3D–G) in *Pkd1*^RC/RC^ kidneys were also significantly reduced by nintedanib treatment. However, in *Pkd1*^RC/RC^ kidneys, nintedanib treatment did not change SMAD3 activity, an important component of TGFβ-signaling pathway and a contributor to renal fibrosis, as suggested by no change in pSMAD3/SMAD3 ratio, when compared with vehicle treatment (Supplemental 4A, B).

### In *Pkd1*KO mice, nintedanib treatment reduced renal cyst growth, but not fibrosis

We also tested the effect of nintedanib in *Pkd1*KO (*Pkd1*^f/f^*Pkhd1*^*Cre*^) mice, an ADPKD model with rapid disease progression and a high rate of cystic epithelial cell proliferation [26, 27].

**Fig. 4:**
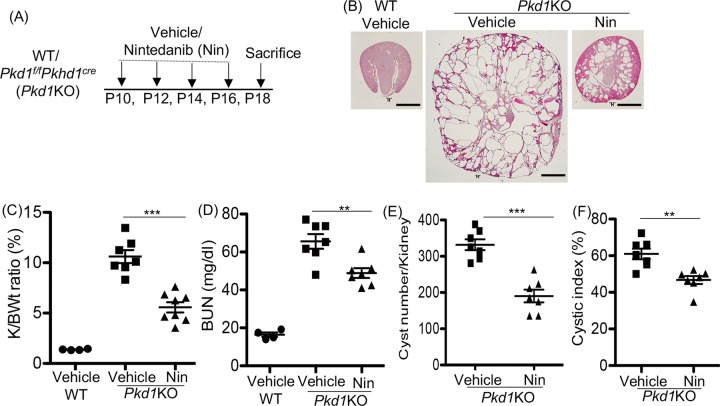
Nintedanib (Nin.) treatment reduced renal cyst growth in Pkd1f/fPkhd1cre (Pkd1KO) mice. **A** Scheme of treatment. WT or *Pkd1*KO mice were treated with vehicle or Nintedanib (20 mg/Kg BWt, IP) on P10, P12, P14, and P16, and sacrificed on P18. **B** H&E staining of kidney sections of WT or *Pkd1KO* mice treated with vehicle or Nin sacrificed at P18. Scale bar = 1 mm. **C** Two kidney to body-weight ratios (%). **D** Plasma blood-urea nitrogen (BUN) levels. **E** Cyst number/kidney, (**F**) Cystic index (%). *P < 0.05, **P < 0.01, ***P < 0.001 by *T*-test.

*Pkd1*KO and WT littermates (*Pkd1*^f/f^ with no cre) were treated with vehicle or nintedanib (20 mg/kg BWt) on postnatal days P10, P12, P14, and P16, and sacrificed on P18 (Fig. 4A). Nintedanib treated *Pkd1*KO mice had smaller kidneys (Fig. 4B), 50% reduction in kidney to body-weight ratio (Fig. 4C), 28% reduction in BUN (Fig. 4D), 40% fewer cysts (Fig. 4E) and 25% smaller percent cyst area in the kidneys (Fig. 4F) when compared with vehicle-treated *Pkd1*KO mice. In WT mice, nintedanib treatment did not alter renal morphology or kidney to body-weight ratio when compared with vehicle treatment (Supplemental 5A–C).

**Fig. 5:**
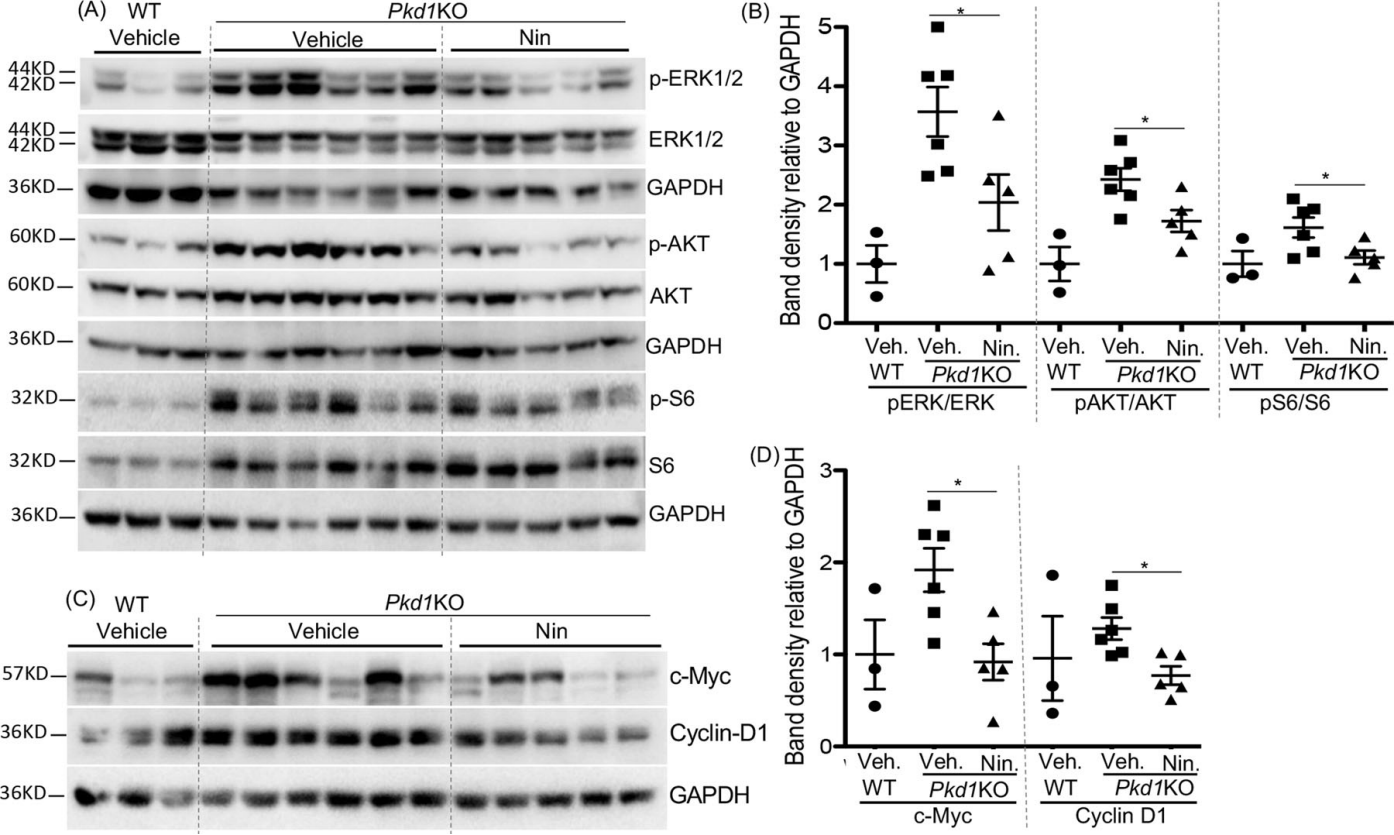
Nintedanib (Nin) treatment reduced proproliferative cell signaling in Pkd1KO mice. **A** Immunoblot of kidney tissue lysate of mice treated with vehicle (Veh.) or Nin., and (**B**) quantitation of band density. **C** Immunoblot of kidney tissue lysate for proproliferative factors and (**D**) quantitation of band density. *P < 0.05, **P < 0. 01, ***P < 0.001 by T-test for Nin vs vehicle-treated *Pkd1*KO mice.

*Pkd1*KO kidneys showed no significant change in FGFR1, PDGFRβ, or VEGFR2 expression compared with WT kidneys (Supplemental 6A, B). However, downstream effectors of RTK cell signaling including ERK, AKT, and mTOR [4] activity, and proproliferative factors such as c-Myc [28] and Cyclin D1 were increased in *Pkd1*KO kidneys and nintedanib treatment significantly reduced their activity or expression compared with vehicle treatment (Fig. 5A–D). Nintedanib treatment did not significantly change YAP, or pSTAT3/STAT3 ratio or αSMA protein levels in *Pkd1*KO kidneys (Supplemental 7A–D). No significant difference was observed in mRNA levels of ECM proteins (data not shown) or pSMAD3/SMAD3 ratio between nintedanib and vehicle treatment in *Pkd1*KO mice (Supplemental 7E, F).

## Discussion

In this study, we show for the first time that nintedanib, an FDA-approved triple RTK inhibitor, significantly reduces ADPKD disease progression. We demonstrated that in vitro, nintedanib reduced human ADPKD renal cystic epithelial cell proliferation and cyst growth, as well as reduced ADPKD renal myofibroblast migration and cell viability. In *Pkd1*^RC/RC^ and *Pkd1*KO mice, nintedanib treatment significantly inhibited renal RTK cell signaling, reduced cystic epithelial cell proliferation and cyst growth, and overall disease progression. Nintedanib treatment also significantly inhibited fibrosis in *Pkd1*^RC/RC^ kidneys.

Aberrant expression or activity of RTKs results in altered intracellular signaling and contributes to the progression of chronic diseases of the lungs, kidneys and liver, and in various cancers [4]. The roles of EGF/EGFR and VEGF/VEGFR in PKD are complicated. EGFR inhibitors, such as EKI-785, EKB-569, and Tesevatinib, and the Src inhibitor SKI-606, which also inhibits the EGFR signaling, have been shown to slow cyst growth in rodent models of ARPKD, ADPKD, and other forms of PKD [7, 9–11, 13]. The role of EGF/EGFR appears to also depend on the postnatal developmental stage because in neonatal ARPKD mice, EGF treatment did not reduce cyst expansion and also caused early death [7]. Increased expression of VEGF in renal cystic epithelial cells and VEGFR2 in endothelial cells has been detected in patients with ADPKD [29]. Moreover, a VEFGA gene polymorphism has been positively correlated with ADPKD progression [30, 31]. Expression of VEGF, VEGFR1, and VEGFR2 has also been shown in Cy/+ rats, an ADPKD rodent model characterized by proximal tubule-derived cysts [12]. Consistently, ribozymes that specifically inhibit VEGFR1 and VEGFR2 mRNA expression significantly reduced renal cyst growth in Cy/+ rats [12], while SU5416, a small-molecule inhibitor of VEGFR2, reduced hepatic cyst growth in Pkd2 KO mice [8]. However, targeting the ligand using anti-VEGF antibody treatment induced severe renal failure and glomerular damage, and increased proximal tubular cyst growth in Cy/+ rats [32]. By contrast, anti-VEGF antibody treatment in cancer patients did not change the size of simple renal cysts [33].

The role of PDGFR and FGFR is not well studied in PKD. PDGF immunoreactivity was detected in cyst-lining epithelium of human kidneys with acquired cystic disease [34, 35] and in ADPKD [35]. Moreover, PDGF treatment stimulated proliferation of ADPKD fibroblasts, but not ADPKD epithelial cells in vitro [35]. Similarly, FGF had a greater effect on the proliferation and intracellular RTK-mediated cell signaling in human ADPKD renal fibroblasts than normal renal fibroblasts [36]. In Pkd1^RC/RC^ kidneys, we found significant increase in PDGFRβ and FGFR1 expression compared with WT kidneys, but VEGFR2 expression remained unchanged. In Pkd1KO kidneys, PDGFRβ, VEGFR2, and FGFR1 levels showed no difference compared to WT littermates. However, known cell signaling mediated by these RTKs was found to be upregulated. PDGFRs, VEGFRs, and FGFRs signal via the SRC kinase and regulate STAT3, AKT, ERK, MAPK, or JNK-mediated proliferative and migratory pathways in tumor cells [18]. These cell-signaling pathways also stimulate renal cyst expansion and inflammation in PKD [9, 11, 23], and could be regulated by multiple mechanisms in the PKD kidney [37]. Hence, our findings that ERK, AKT, mTOR and STAT3 activities are elevated in PKD and were significantly reduced by nintedanib treatment suggests that the activities of RTKs (PDGFR, VEGFR and FGFR) are important in the Pkd1^RC/RC^ and Pkd1KO kidneys. Consistent with reduced intracellular RTK-mediated cell signaling, nintedanib-treatment reduced human ADPKD epithelial cell proliferation and cyst growth in vitro, and reduced cystic epithelial cell proliferation and renal cyst growth in both Pkd1^RC/RC^ and Pkd1KO models in our study. While the percent cystic area in the nintedanib treatment group was significantly reduced in both Pkd1^RC/RC^ and Pkd1KO kidneys, the cyst numbers were reduced only in Pkd1KO kidneys. This could be because at three months of age when nintedanib treatment was started in Pkd1^RC/RC^ mice, their kidneys already had small cysts. By comparison, when nintedanib treatment was started at postnatal day 10 in the Pkd1KO mice, their renal cysts were very small or not detectable [38].

In PKD kidneys, increased ECM production, reduced degradation, and changes in its composition lead to fibrosis, and subsequent loss of renal function [3, 39–42]. Nintedanib has been demonstrated to be a very effective antifibrotic drug in preclinical and clinical studies [14–16, 19–21], including a CKD mouse model [22]. In our in vitro studies, nintedanib treatment significantly inhibited cell proliferation and migration of cultured human ADPKD renal myofibroblasts and significantly reduced renal fibrosis in the *Pkd1*^*RC/RC*^ mouse. However, nintedanib had no effect in the *Pkd1*KO mouse. This could be because disease progression in the *Pkd1*^*RC/RC*^ model is relatively slow and fibrosis becomes evident in the adult mice by 4–5 months of age. The *Pkd1*KO model, on the other hand, is a preweaning, rapidly progressing model, which may be suitable to study early increases in the myofibroblast population and ECM deposition, but perhaps not mature fibrosis. However, it should also be noted that in the *Pkd1*^*RC/RC*^ mouse, all cells, including the myofibroblasts, are *Pkd1* hypomorphs, while in the *Pkd1*KO mouse, *Pkd1* deletion only occurs in the collecting ducts. Nintedanib is known to reduce TGFβ-induced fibroblast-to-myofibroblast differentiation, migration and proliferation of tumor stromal fibroblasts, and lung and renal fibroblasts [14, 43]. While nintedanib treatment in our study significantly reduced renal fibrosis in *Pkd1*^*RC/RC*^ mice, it did not affect activity of SMAD3, an important component of TGFβ signaling.

In conclusion, these findings demonstrate that nintedanib, a triple RTK inhibitor, reduces proliferation and in vitro cyst formation of human ADPKD cystic cells. Nintedanib also inhibits epithelial cell proliferation and cyst growth in slowly progressing adult and rapidly progressing prenatal models of ADPKD. As such, we propose that nintedanib could be a useful pharmacological approach to slow ADPKD progression in patients.

## Materials and methods

### In vivo studies

#### Mouse models

(A) The *Pkd1*^RC/RC^ mouse, a slowly progressive orthologous ADPKD model that contains a temperature-sensitive folding hypomorphic mutation (R3277C) in polycystin-1 [44, 45] and wild-type (WT, *Pkd1*^*+/+*^) mice, ﻿was maintained on BALB/c background. (B) The *Pkd1*^*f/f*^*Pkhd1*^*Cre*^ (*Pkd1*KO) mouse, a rapidly progressive ADPKD model caused by a mostly collecting duct-specific *Pkd1* gene deletion [26, 27, 38, 46], and WT (*Pkd1*^*f/f*^) mouse littermates without Cre, was on C57Bl/6 J background.

#### Nintedanib study

Nintedanib (Selleck Chemicals, TX) was administered at 20 mg/Kg BWt dose on alternate days by intraperitoneal injections. This dose was well tolerated in adult WT C57/Bl6J mice in our pilot studies (data not shown). *Pkd1*^RC/RC^ mice were treated from 3 to 5 months of age and sacrificed and *Pkd1*KO mice from postnatal day P10 to P16 and sacrificed on P18. Mouse sample size for the two studies was selected based on our preliminary data from pilot studies (data not shown). Mice were not randomized, instead, littermates were assigned either to vehicle or to nintedanib-treatment groups. For some analyses such as immunoblot and immunostaining, tissues of all mice were not analyzed, instead, few samples in each study group were randomly chosen. All animal studies were performed according to protocols approved by the University of Kansas Medical Center’s (KUMC) Institutional Animal Care and Use Committee.

#### Quantification of cysts and tissue fibrosis

Hematoxylin and eosin staining was performed on kidney tissue sections (5-µm thick) and imaged using Nikon 80i upright microscope (Tokyo, Japan). Quantification of cyst number, area, and total kidney area was performed using ImageJ (Fiji, Madison, WI, USA) by an observer blinded to sample identity. Picro sirius red staining (Polysciences, Warrington, PA, USA) was performed for kidney tissue sections following the manufacturer’s protocol. Stained tissue sections were imaged under polarized light and the amount of polarizable collagen per unit area was quantified.

#### Blood-urea nitrogen (BUN)

BUN levels were measured in serum as described previously [27] using the QuantiChrom Urea Assay Kit from BioAssay Systems (Hayward, CA, USA).

#### Western blot

Mouse kidneys were homogenized in SDS Laemmli buffer and loaded onto 10% SDS-polyacrylamide agarose electrophoresis gels essentially as described previously [27, 47]. Primary antibodies pERK1/2 (9101 S), pAKT (4060 S), pSTAT3 (9145 S), STAT3 (9139 S), cyclinD1 (2978 S), S6 (2317 S), pS6 (4858 S), PDGFRβ (3169), VEGFR2 (9698), and FGFR1 (9740) from Cell signaling (Danvers, MA, USA); YAP (SC-101199), GAPDH (SC-32233), ERK1/2 (SC-94), cMyc (SC-40) from Santa Cruz Biotechnology, Inc. (Dallas, TX, USA), and α-SMA (ab5694; Abcam, Cambridge, MA, USA); and secondary antibodies, anti-mouse (P0447) and anti-rabbit (P0448) from Dako (Santa Clara, CA, USA) and ECL reagent from Amersham (GE Health care, Buckinghamshire, UK) were used.

### Immunohistochemistry/immunofluorescence (IHC/IF)

Fixed and paraffin tissue sections were processed as described before [48]. Primary antibodies, α-SMA (ab5694) and FSP1 (ab27957) from Abcam (Cambridge, MA, USA), Ki67 (94495) from Cell Signaling, (Danvers, MA, USA), Vimentin (SC7557) from Santa Cruz Biotechnology, Inc. (Dallas, TX, USA), and Pan-cytokeratin (F0397) from (MilliporeSigma, St. Louis, MO) were used. For IHC, secondary antibody application was followed by incubation with Streptavidin HRP conjugate (Invitrogen, Carlsbad, CA, USA), and DAB (Vector Laboratories, Burlingame, CA, USA), and counterstained with Harris Haematoxylin, dehydrated, and mounted with Permount (Fisher Scientific, Waltham, MA, USA). For IF, goat anti-Rabbit IgG fluor and Goat anti-mouse IgG Texas red (Invitrogen, Carlsbad, CA, USA), secondary antibodies were applied, incubated, washed and stained with DAPI, and mounted with Flour-G (Invitrogen, Carlsbad, CA, USA). Images were captured using a Nikon 80i upright microscope (Tokyo, Japan).

#### Quantitative real-time PCR

From whole kidney tissue lysate, RNA was isolated using the trizol method (Ambion, Austin, TX, USA) and cDNA was prepared using High capacity CDNA reverse transcription kit from Applied Biosystems (Foster City, CA, USA) and QRTPCR performed using power SYBR Green PCR master mix Applied Biosystems (Foster City, CA, USA) following the manufacturer’s protocol. Primer sequences are shown before [26].

### In vitro studies

#### Primary culture human ADPKD cells

Primary culture human renal ADPKD cystic epithelial cells and normal human kidney (NHK) cells [26] were provided by the KUMC PKD Biomarkers and Biomaterials Core, a part of the national PKD Research Resource Consortium (PKD-RRC). Primary culture human myofibroblasts from ADPKD kidneys were isolated using a standard protocol described before [49] and cells were used in their first passage. ADPKD kidneys and NHK were obtained by this core from the surgery department at KUMC by KU Cancer Center’s Biospecimen Resource Core and hospitals participating in Tissue Donation Program of PKD Foundation (Kansas City, MO). These deidentified tissue samples have only alphanumeric codes and basic clinical information, and have IRB approval (IRB #5929; approval date 8/25/2020) to consent patients for “medical discard” nephrectomy (routine care) samples to use for research. Mycoplasma contamination was monitored by DAPI staining and confirmed using a mycoplasma testing kit (PCR method).

#### In vitro cystogenesis assay

In vitro 3D cyst-growth assay was performed as before [38] using ADPKD epithelial cells. On day 4, when cysts were evident, nintedanib (1.5 µM) or vehicle (DMSO 1 µl/ml) was added for 12 days. Cysts were photographed and cyst diameter (≥30 μM) measured using Image-Pro Premier software.

#### Fibroblast to myofibroblast differentiation

NRK-49F (ATCC^®^ CRL-1570, Manassas, VA, USA) rat renal fibroblast cells grown in DMEM media, when 50% confluent, were treated with 2 ng/ml of TGFβ for 48 h, lysed, and αSMA levels measured.

#### Migration (wound-closure) assay

NRK-49F or human ADPKD renal myofibroblasts were seeded in 6-well plates, grown in DME/F-12 media, and wound closure assay performed as described earlier [26].

#### Cell proliferation and cell viability

To assess cell proliferation, M-1 mouse renal cortical collecting duct cell (#CRL-2038, American Type Culture Collection, Manassas, VA) and human primary culture NHK and ADPKD epithelial cells grown on glass coverslips were treated with vehicle or Nintedanib for 24 h and treated with BrdU (#10280879001, MilliporeSigma, Burlington, MA) (3 μg/mL) for 3 h and cell proliferation measured as described [50]. To measure cell viability, cells were seeded in 24-well plates and MTT assay performed as described [51].

#### Statistics

Values were expressed as mean ± standard error for in vivo, and mean ± standard deviation for in vitro studies. Data were analyzed by two-tailed unpaired T-test with Welch’s correction, or one-way ANOVA followed by Dunnett’s multiple-comparison test using GraphPad Prism software (Version 5.0d). *P* ≤ 0.05 was considered significant.

## Supplementary information

Supplemental Figure legends

Supplemental Figure 1

Supplemental Figure 2

Supplemental Figure 3

Supplemental Figure 4

Supplemental Figure 5

Supplemental Figure 6

Supplemental Figure 7

## Data Availability

The data and materials generated from this study are available upon reasonable request from the corresponding author.
