# Supplementary material for: The tyrosine-kinase inhibitor Nintedanib ameliorates autosomal-dominant polycystic kidney disease
Source: Cell Death Dis. 2021 Oct 14;12(10):947. doi: 10.1038/s41419-021-04248-9 (PMC8517027; doi:10.1038/s41419-021-04248-9)
Supplement: Supplementary file 1 — Supplemental Figure legends [file 41419_2021_4248_MOESM1_ESM.docx]

**Supplemental Material**

**The Tyrosine Kinase Inhibitor Nintedanib Ameliorates Autosomal Dominant Polycystic Kidney Disease**

Abeda Jamadar^a,b^, Sreenath M Suma^a,b^, Sijo Mathew^c^, Timothy A Fields^d^,

Darren P Wallace^a,b^, James P Calvet^a,e^ and Reena Rao^a,b^ *****

^a^ The Jared Grantham Kidney Institute, University of Kansas Medical Center, Kansas City, KS. ^b^ Department of Medicine, University of Kansas Medical Center, Kansas City, KS. ^c^ Department of Pharmaceutical Sciences, School of Pharmacy, North Dakota State University, Fargo, ND. ^d^ Department of Pathology and Laboratory Medicine, University of Kansas Medical Center, Kansas City, KS. ^e^ Department of Biochemistry and Molecular Biology, University of Kansas Medical Center, Kansas City, KS.

**Correspondence:**

***** Reena Rao (PhD E-mail: [rrao@kumc.edu](mailto:rrao@kumc.edu))

**Supplemental Figure Legends:**

**Supplemental 1. Effect of nintedanib on renal epithelial cells and myofibroblasts:** (A) BrdU incorporation assay showing effect of nintedanib treatment (1.5µM for 24h) in primary culture normal human kidney (NHK) epithelial cells. n=3 biological replicates (patient samples) and 5 technical replicates each, or (B) M-1 mouse collecting duct cells. (C) Immunoblot of human ADPKD renal myofibroblasts cells incubated with nintedanib (1.5μM) for 48h and (D) Quantitation of band density. * P<0.05, ** P<0.01, ns=not significant by T-test.

**Supplemental 2. Expression of TK receptors in WT and *Pkd1*^RC/RC^ kidneys:** (A) Immunoblot for TK receptors in WT and *Pkd1*^RC/RC^ mouse kidneys and (B) Quantitation of band density. (C) QRTPCR for PDGFRα and PDGFRβ in WT and *Pkd1*^RC/RC^ mouse kidneys. * P<0.05, ** P<0.01 and ns = not significant by T-test.

**Supplemental 3. Effect of nintedanib on WT kidneys:** (A) H&E staining of kidney sections of WT mice (littermates of *Pkd1^RC/RC^* mice) treated with vehicle or nintedanib. Scale bar=1mm. (B) High magnification images of H&E stained sections. (scale bar=50µM). (C) Two kidney to body weight ratio (%). (D) Immunostaining for KI-67 in mouse kidney sections. (scale bar =50µM) ‘ns’= not significant by T-test.

**Supplemental 4. Effect of nintedanib on C-Myc, Cyclin-D1 levels and SMAD3 activity in *Pkd1*^RC/RC^ kidneys:** (A) Immunoblot on WT and *Pkd1*^RC/RC^ kidney tissue lysate and (B) Quantitation of band density for C-Myc and Cyclin D1 and pSMAD/SMA3 ratio (fold change). ns=not significant by T-test.

**Supplemental 5.** **Effect of nintedanib on WT kidneys:** (A) H&E staining of kidney sections of WT mice (littermates of *Pkd1*KO mice) treated with vehicle or nintedanib. Scale bar=1mm. (B) Two kidney to body weight ratio (%). (C) H&E stained sections. (scale bar=50µM). ns=not significant by T-test.

**Supplemental 6.** **RTK receptors in WT and *Pkd1*KO kidneys:** (A) Immunoblot for RTK receptors in WT and *Pkd1*KO mouse kidneys and (B) Quantitation of band density. ns=not significant by T-test.

**Supplemental 7:** **Effect of Nintedanib in *Pkd1*KO mouse kidneys:**

(A) Immunoblot on WT and *Pkd1*KO kidney tissue lysate and (B) Quantitation of band density for YAP, (C) pSTAT3 to STAT3 ratio and (D) αSMA. (E) Immunoblot and (F) Quantitation of band density for pSMAD/SMA3 ratio. ns=not significant by T-test.
