# Supplementary figures and images for: The tyrosine-kinase inhibitor Nintedanib ameliorates autosomal-dominant polycystic kidney disease
Source: Cell Death Dis. 2021 Oct 14;12(10):947. doi: 10.1038/s41419-021-04248-9 (PMC8517027; doi:10.1038/s41419-021-04248-9)

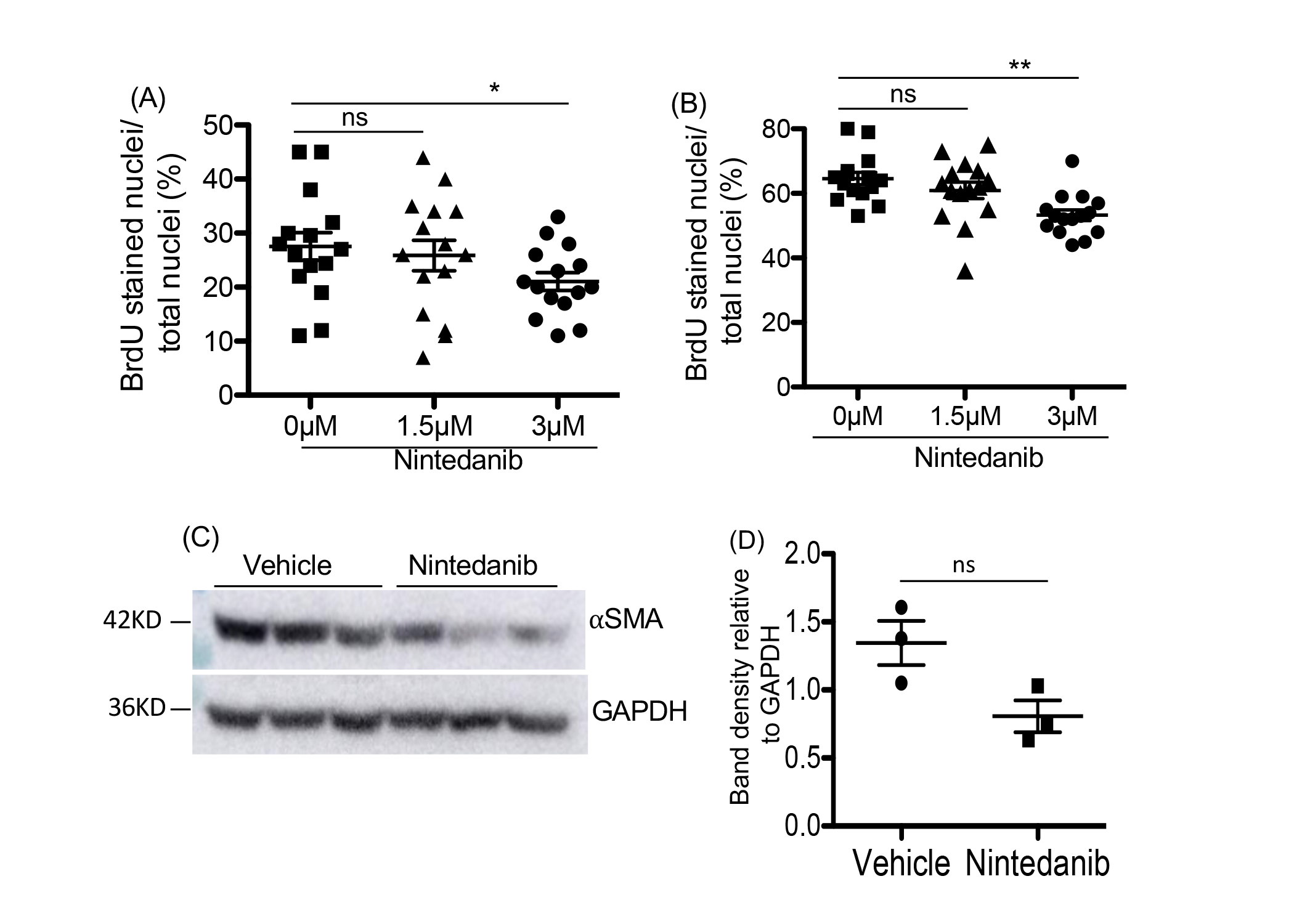

Supplement: Supplementary file 2 — Supplemental Figure 1 [file 41419_2021_4248_MOESM2_ESM.tif]

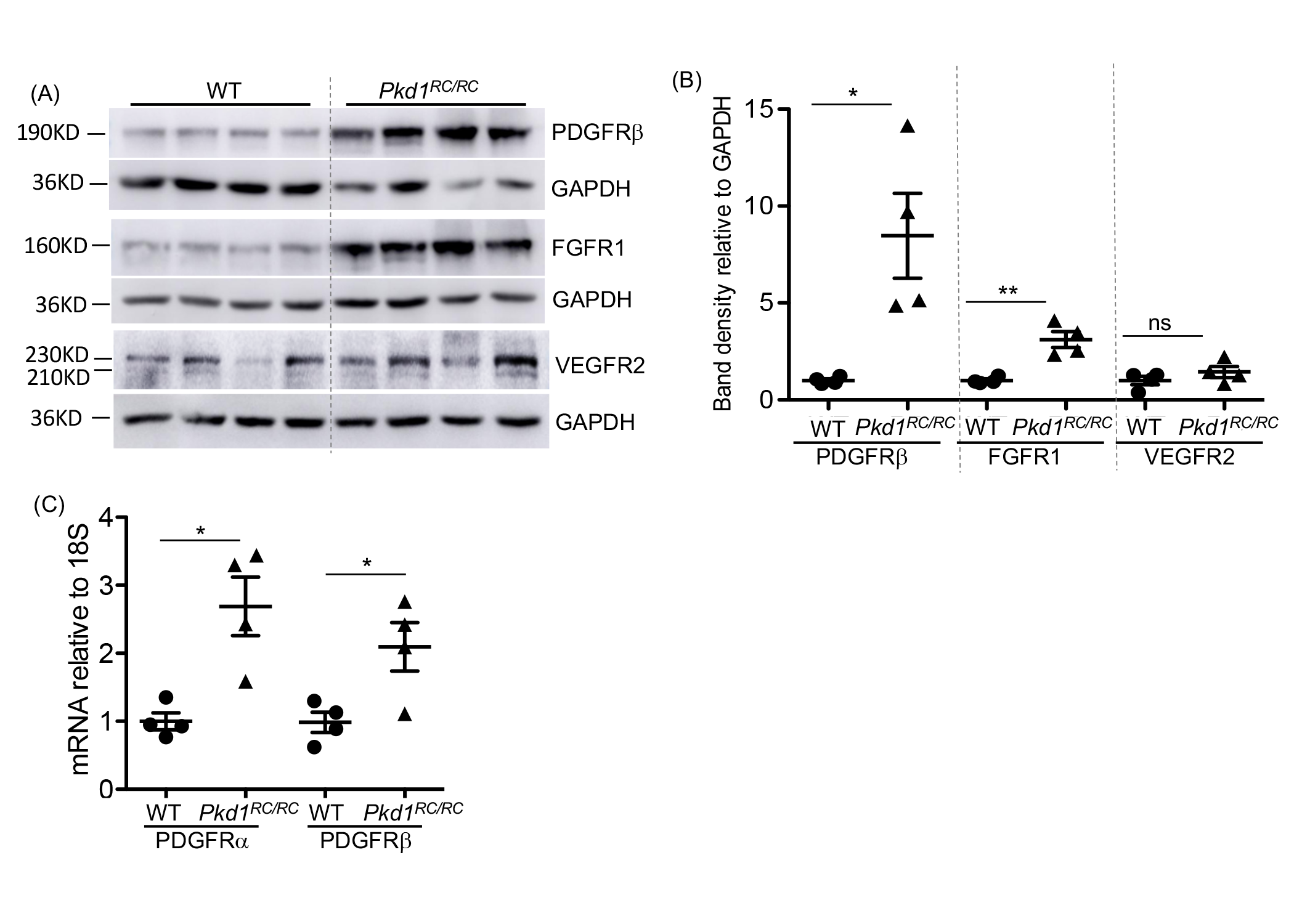

Supplement: Supplementary file 3 — Supplemental Figure 2 [file 41419_2021_4248_MOESM3_ESM.tif]

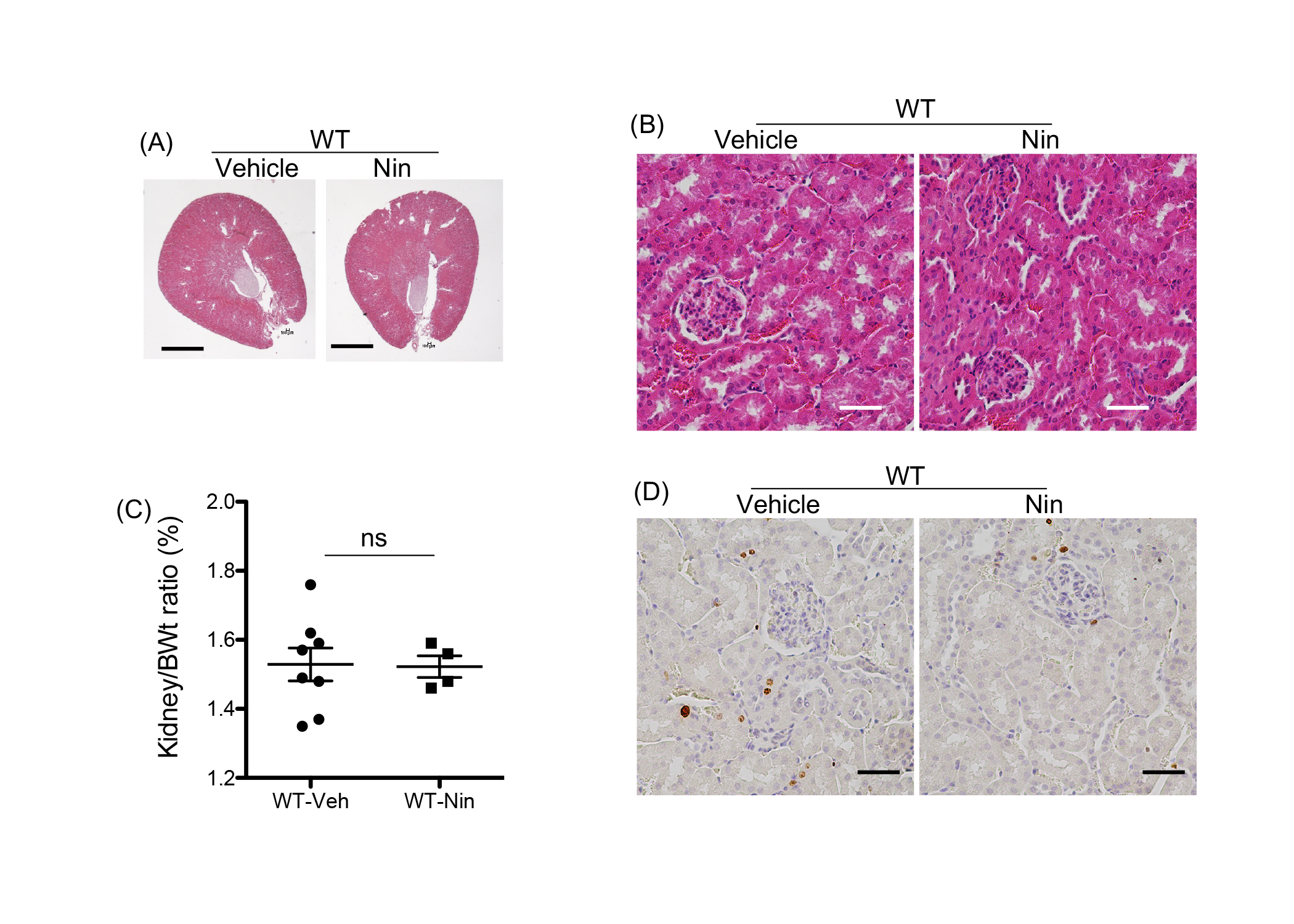

Supplement: Supplementary file 4 — Supplemental Figure 3 [file 41419_2021_4248_MOESM4_ESM.tif]

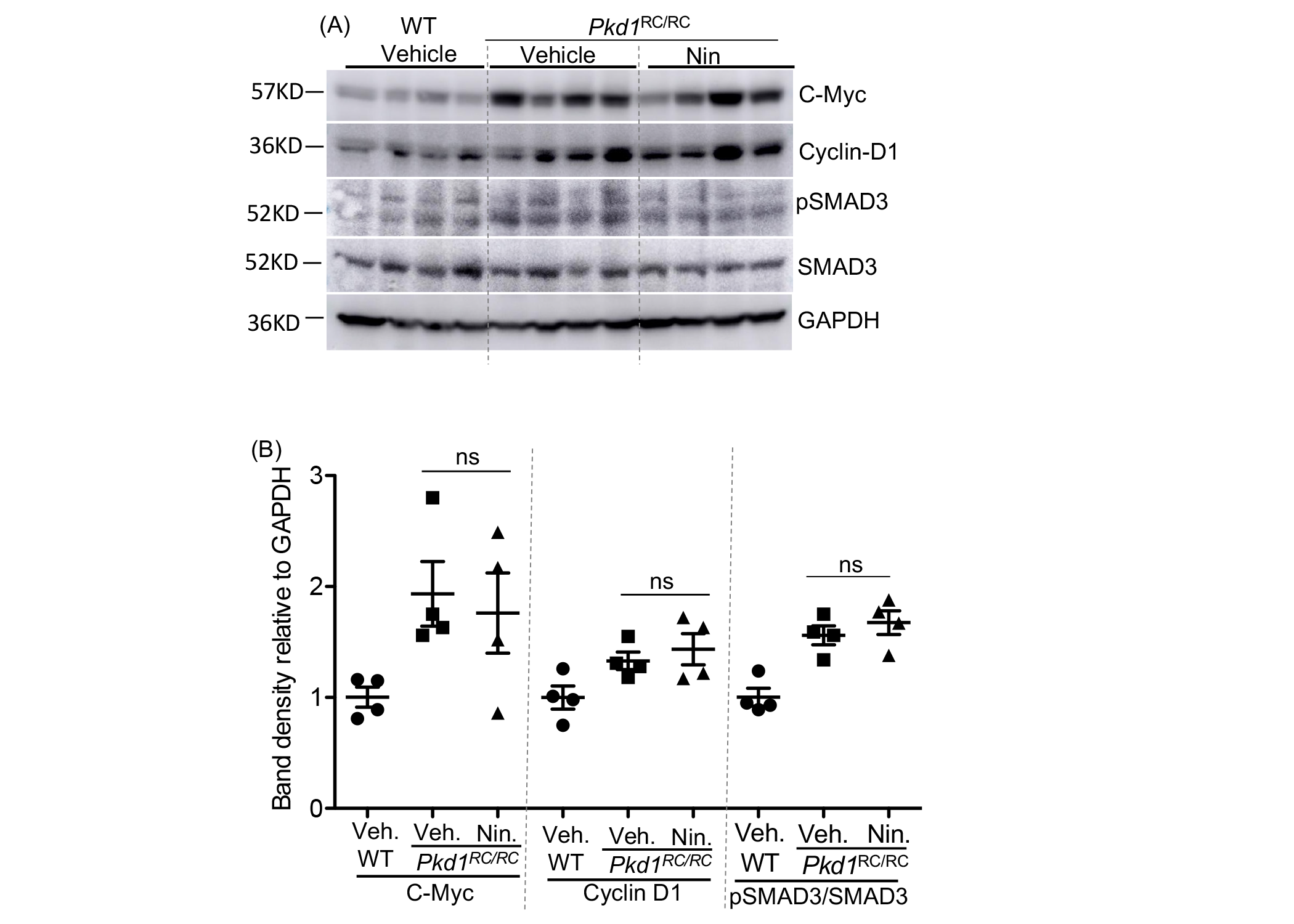

Supplement: Supplementary file 5 — Supplemental Figure 4 [file 41419_2021_4248_MOESM5_ESM.tif]

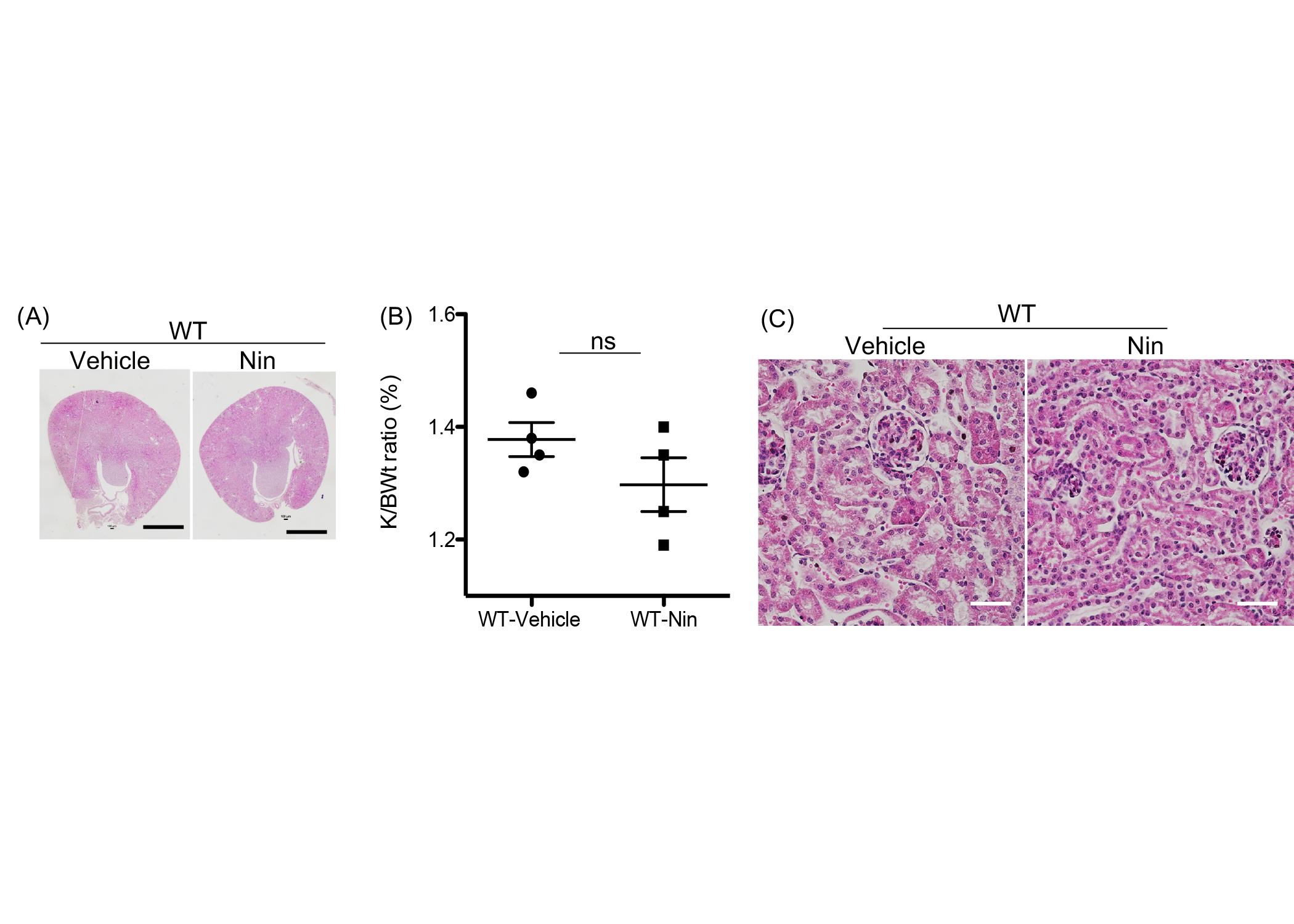

Supplement: Supplementary file 6 — Supplemental Figure 5 [file 41419_2021_4248_MOESM6_ESM.tif]

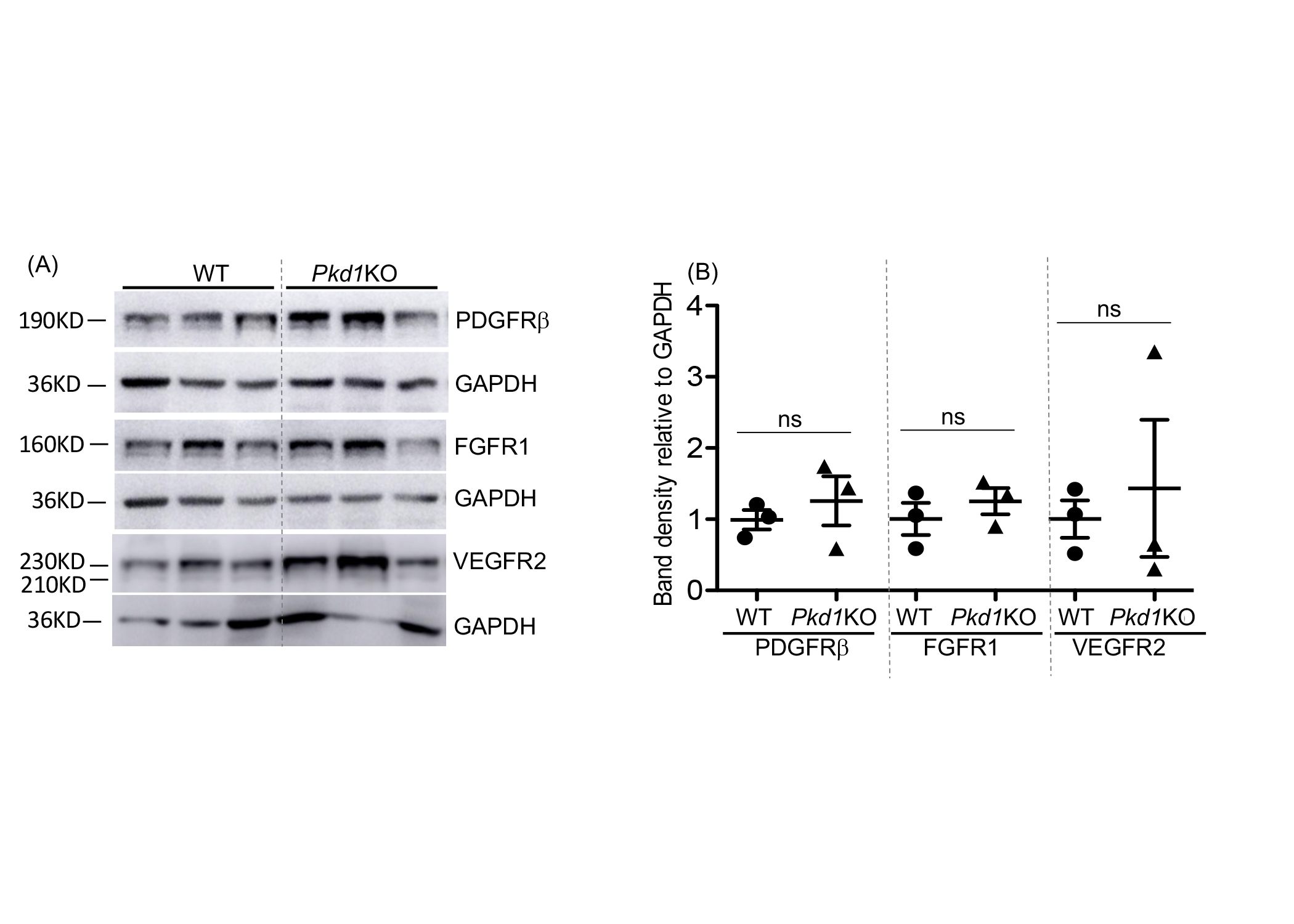

Supplement: Supplementary file 7 — Supplemental Figure 6 [file 41419_2021_4248_MOESM7_ESM.tif]

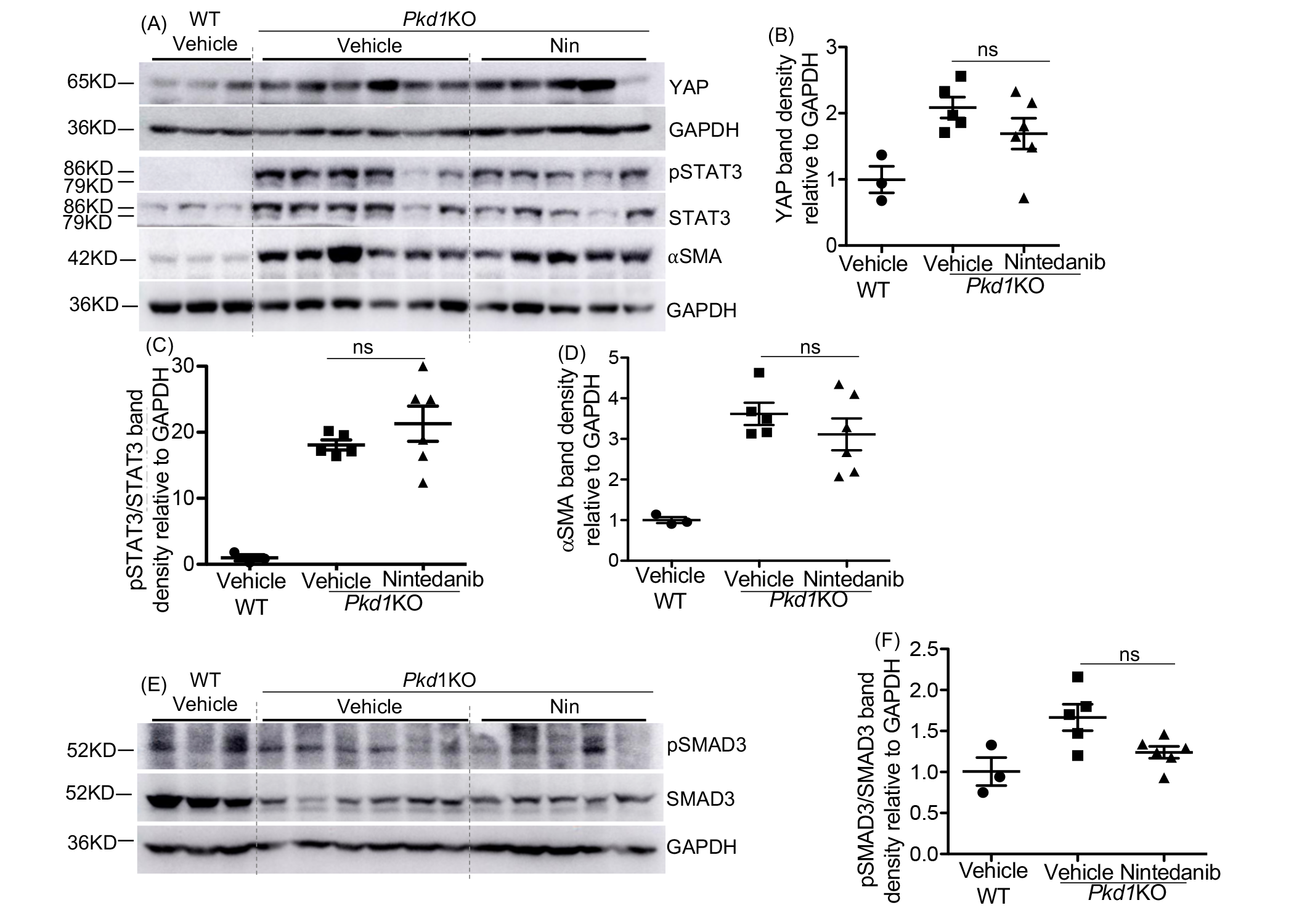

Supplement: Supplementary file 8 — Supplemental Figure 7 [file 41419_2021_4248_MOESM8_ESM.tif]
